# Hydrogel Applications for Cultural Heritage Protection: Emphasis on Antifungal Efficacy and Emerging Research Directions

**DOI:** 10.3390/gels11080606

**Published:** 2025-08-02

**Authors:** Meijun Chen, Shunyu Xiang, Huan Tang

**Affiliations:** 1 Key Scientific Research Base of Pest and Mold Control of Heritage Collection, Chongqing China Three Gorges Museum, State Administration of Cultural Heritage, Chongqing 400015, China; cmjecho@163.com; 2 College of Plant Protection, Southwest University, Chongqing 400715, China

**Keywords:** cultural heritage preservation, antifungal hydrogels, biodeterioration prevention, stimuli-responsive materials, nanoparticle-infused hydrogels

## Abstract

Hydrogels, characterized by their high water content, tunable mechanical properties, and excellent biocompatibility, have emerged as a promising material platform for the preservation of cultural heritage. Their unique physicochemical features enable non-invasive and adaptable solutions for environmental regulation, structural stabilization, and antifungal protection. This review provides a comprehensive overview of recent progress in hydrogel-based strategies specifically developed for the conservation of cultural relics, with a particular focus on antifungal performance—an essential factor in preventing biodeterioration. Current hydrogel systems, composed of natural or synthetic polymer networks integrated with antifungal agents, demonstrate the ability to suppress fungal growth, regulate humidity, alleviate mechanical stress, and ensure minimal damage to artifacts during application. This review also highlights future research directions, such as the application prospects of novel materials, including stimuli-responsive hydrogels and self-dissolving hydrogels. As an early exploration of the use of hydrogels in antifungal protection and broader cultural heritage conservation, this work is expected to promote the wider application of this emerging technology, contributing to the effective preservation and long-term transmission of cultural heritage worldwide.

## 1. Introduction

Cultural relics are vital carriers of human history, art, and science, yet they face continuous deterioration over time due to physical damage, chemical corrosion, and biological invasion [1,2]. Among these threats, fungal biodeterioration is particularly destructive and widespread [3]. Fungi are ubiquitous in the environment and can rapidly colonize artifact surfaces under favorable conditions, such as high humidity, moderate temperature, adequate oxygen, and the presence of organic nutrients [4]. Both organic and inorganic relics are susceptible to fungal degradation [4]. Organic relics, such as paper, textiles, wood, and leather, are especially vulnerable due to their composition of natural polymers like cellulose, lignin, and proteins, which serve as ideal nutrient sources for fungal growth [5,6]. Once contaminated, fungal hyphae can penetrate the internal structure of the artifacts, causing fiber breakage and microscopic structural damage [7]. Additionally, metabolic by-products such as enzymes and organic acids can lead to discoloration, staining, and surface degradation [7]. Inorganic relics, including stone carvings and grotto sculptures, though composed mainly of mineral materials, are not immune to fungal damage [6,8,9,10]. When exposed to the open environment, fungi growing on stone surfaces can secrete acidic compounds that react with minerals, leading to surface etching, erosion, and eventual material loss.

**Figure 1:**
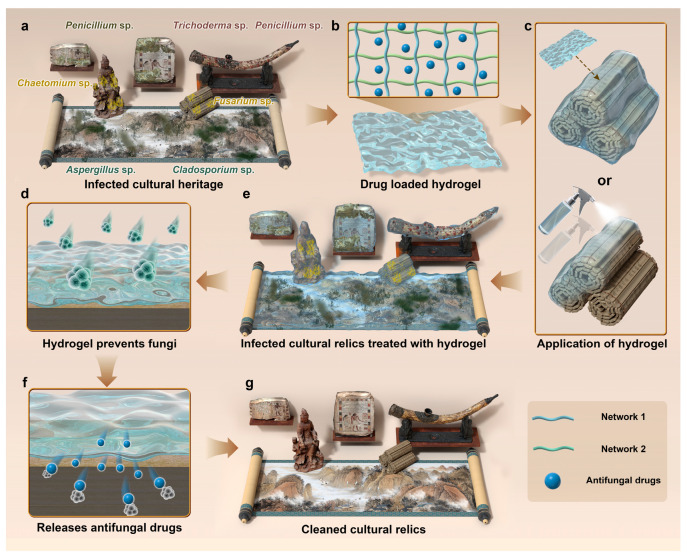
This schematic diagram illustrates the use of hydrogel for preventing and controlling fungal infections on the surface of cultural heritage. The cultural relics in this figure are arranged in three rows. (**a**–**c**) has three items, which, from left to right, are a stone, mural, and bone relics. (**d**,**e**) contains two wooden artifacts, and (**f**,**g**) features a paper-based relic.

Conventional techniques for cultural heritage preservation face notable limitations in combating fungal biodeterioration, falling short of the increasingly rigorous standards required for modern conservation [11,12]. Physical preventive measures, such as improving ventilation and regulating temperature and humidity, can partially inhibit fungal growth but are insufficient to fully prevent its proliferation and spread [13,14,15]. Chemical fungicides, while offering some degree of efficacy, carry the risk of secondary contamination and potential damage to the artifacts themselves [16,17,18]. Moreover, prolonged use may promote the development of fungal resistance [16]. In recent years, hydrogels, novel polymeric materials characterized by their three-dimensional network structures and unique physicochemical properties, have gained significant attention across fields such as biomedicine, drug delivery, and agriculture [19,20,21,22,23]. Their application in cultural heritage conservation and restoration has shown great promise, particularly in addressing fungal-related deterioration [24,25,26,27,28,29,30]. Owing to their superior water absorption, moisture retention, biocompatibility, and customizable design, hydrogels can create a stable microenvironment that mitigates environmental stressors affecting cultural heritage [29]. More specifically, the antifungal efficacy of hydrogels in cultural heritage conservation primarily relies on their excellent controlled-release capability and interfacial adaptability. Their three-dimensional polymer network allows for the loading of antifungal agents and facilitates their sustained release via diffusion or in response to environmental stimuli such as pH or temperature, effectively inhibiting fungal growth. In addition, hydrogels can act as a physical barrier to prevent recontamination by microorganisms, thereby reducing the risk of secondary damage to the artifact. Owing to their superior moisture retention and moldability, hydrogels can closely conform to the surface of cultural objects, enhancing the localized effectiveness of the antifungal agents (Figure 1) [31]. However, current research on the systematic application of hydrogels in heritage conservation remains limited. This review aims to summarize recent progress in the use of hydrogel-based technologies for fungal control in cultural heritage, with the goal of advancing their practical implementation and fostering innovation in heritage science.

Currently, research on the application of hydrogels in the antifungal treatment of cultural heritage is relatively limited. In April 2025, we conducted a search using the keywords “Hydrogel,” “Cultural relics or Cultural heritage,” and “Fungi” on two major databases, Google Scholar and Web of Science [32]. A total of 326 relevant records were retrieved, including research articles, reviews, conference papers, and patents, with no restriction on publication date. We selected the most relevant literature for focused discussion.

## 2. Fungal Species and Deterioration Mechanisms in Cultural Heritage

Among the various prokaryotic and eukaryotic microorganisms capable of colonizing and biodeteriorating materials, fungi are particularly prominent in built environments, with a long history of involvement in the contamination and deterioration of man-made products and cultural heritages [33]. They are considered the primary colonizers of surfaces such as stone, mortar, and plaster. Commonly identified fungal genera on a wide range of deteriorated heritage materials, including textiles, canvas paintings, wood, paper, parchment, stone, wall paintings, metals, and audiovisual media, include *Alternaria*, *Aspergillus*, *Cladosporium*, *Fusarium*, *Penicillium*, and *Trichoderma*, with the phylum Ascomycota being the dominant group [33]. The destructive potential of fungi lies in their ability to utilize diverse carbon sources and secrete various extracellular degradative enzymes that act on both natural and synthetic polymers, making nearly all carbon-containing substances susceptible to fungal attack, especially organic-rich materials, such as wood, paper, leather, and textiles [33]. In addition to enzymes, fungi also release a variety of extracellular metabolites, such as organic acids, siderophores, polyphenols, and polysaccharides, which can significantly alter the geochemical properties of their microenvironment and further contribute to the physical and chemical deterioration of metals, alloys, rocks, mineral-based substrates, ceramics, and plaster [33]. A systematic summary of the major fungal taxa commonly found in cultural heritage and their primary biodeterioration pathways is presented in Table 1.

## 3. Hydrogels for Paper Relic Protection

Paper-based cultural heritages carry profound historical, cultural, and artistic value [43], yet their organic composition—primarily cellulose and hemicellulose—renders them highly susceptible to fungal contamination [7,44]. Under favorable conditions of temperature and humidity, common fungi such as *Trichoderma* [39,45], *Aspergillus* [36,45], and *Penicillium* secrete cellulolytic enzymes that hydrolyze cellulose fibers into simple sugars, fueling further fungal proliferation. Residual dust, grease, and traditional adhesives (e.g., starch paste and animal glue) on the surface of paper artifacts also serve as rich nutrient sources. During metabolism, fungi release organic acids that penetrate the paper matrix, accelerating hydrolysis, reducing polymer chain length, and weakening the mechanical integrity of the material [46]. Moreover, fungal colonization leads to visible mold stains and spore deposition that not only obscure inscriptions and artwork but also infiltrate the fiber structure, making remediation extremely challenging [33]. In damp and acidic environments, these degradation processes are significantly intensified, posing a serious threat to the preservation and structural integrity of paper-based heritage materials.

In the conservation of paper-based cultural heritages, preventive protection is as critical as the cleaning and restoration of items already affected by fungal contamination [47,48]. To mitigate biodeterioration risks, antifungal treatments should be applied proactively, prior to visible fungal growth, alongside environmental control measures such as temperature and humidity regulation in museum settings [48,49]. Traditional antifungal agents containing metal ions, while effective to some extent, may accumulate within the microporous structure of paper and pose long-term chemical risks to the artifact [50,51,52,53]. Recently, various types of hydrogels, such as cellulose-based hydrogels, polyacrylic acid hydrogels, gellan gum hydrogels [54], agar hydrogels [55], PVA/PVP semi-interpenetrating network (semi-IPN) hydrogels [56], and dual-network PVA hydrogels [57], have been successfully employed for surface cleaning of artworks. For example, Li et al. developed a gellan gum-based hydrogel incorporating 0.4% (*w*/*w*) Na_2_B_8_O_13_·4H_2_O to inhibit fungal growth on paper artifacts [58]. Experimental results demonstrated a significant reduction in the intensity of peaks corresponding to cellulose degradation products in high-performance liquid chromatography analyses following treatment. On artificially aged Xuan paper, the surface pH increased from 3.0 ± 0.2 to 5.0 ± 0.3, while on Whatman filter paper, it rose from 3.5 ± 0.2 to 5.3 ± 0.3, indicating effective removal of acidic compounds generated through hydrolytic and oxidative degradation. Furthermore, Fourier-transform infrared spectroscopy analysis revealed a marked decrease in the intensity of carbonyl absorption bands within the 1800–1500 cm^−1^ region after treatment, further confirming a reduction in degradation products. Comparative studies showed that traditional aqueous immersion caused water uptake in Xuan paper exceeding 800%, whereas gel treatment limited water absorption to approximately 70%, effectively minimizing fiber swelling and structural deformation. Colorimetric analysis indicated negligible color changes post-treatment (ΔE ≈ 0.5), and no detectable gel residues were found using HPLC, confirming the method’s safety and reversibility. These findings highlight the superior cleaning efficiency, pH regulation, and antifungal properties of the modified gellan hydrogel, underscoring its significant potential for preventive conservation of paper-based cultural heritage.

Based on their mechanical properties and application scenarios, these hydrogels can be broadly categorized into soft gels (SGs, with elastic moduli ranging from a few hundred Pa to tens of Kpa) and rigid gels (RGs, with elastic moduli from hundreds of Kpa to MPa) [59,60]. SGs, such as PVA–borate hydrogels, exhibit good adhesiveness and low elastic modulus, with a texture resembling modeling clay. Their ability to conform closely to textured surfaces is a key advantage in conservation applications. Du et al. employed a poly (vinyl alcohol)/poly (*N*-(2-hydroxyethyl) acrylamide) (PVA/PHEAA) hydrogel, characterized by excellent physical properties and appropriate stiffness, as a model system to systematically investigate the interfacial adhesion between the hydrogel and Xuan paper. A range of analytical techniques was utilized to assess the cleaning performance. The results demonstrated that when the interfacial adhesion energy was below 4 J/m^2^, the hydrogel was capable of achieving effective cleaning while ensuring the structural integrity and safety of the cultural artifact [61]. Despite their favorable surface conformity, SGs present several challenges in practical use. For example, excessive adhesion may lead to mechanical damage to fragile objects or leave residual gel on the surface after cleaning. In contrast, RGs possess higher elastic modulus and structural stability, leaving minimal to no residue post-cleaning. For example, a comparative study demonstrated that both agarose and gellan gum hydrogels effectively removed fungal stains from the surface of paper-based artifacts, exhibiting distinct pH-responsive antifungal activities. Specifically, the gellan gum hydrogel showed a pronounced pH-dependent fungal stain removal capability, achieving a removal rate of up to 100% at pH 6 [62]. These findings suggest their potential as adaptable solutions for cultural heritage conservation under varying environmental conditions. Nonetheless, the limited conformability of RGs to rough or irregular surfaces significantly constrains their practical applicability in cultural heritage conservation.

In summary, although hydrogels have made significant progress in the preservation of paper-based cultural heritages, their application in artwork cleaning still faces several challenges. Most hydrogels exhibit low mechanical strength, making them fragile during use and difficult to remove completely after cleaning. In many cases, removal still relies heavily on manual mechanical methods, which may pose risks to delicate surfaces. Current cleaning hydrogels primarily focus on the controlled release of cleaning solvents (such as water or nanoemulsions), while systematic optimization of the gel matrix itself remains limited. Additionally, adhesion between the hydrogel and paper surfaces may damage paper fibers, compromising the integrity and long-term preservation of the artifact. Therefore, the development of hydrogel systems with improved mechanical stability, controlled-release capability, and minimal material interference represents a key direction for future research in cultural heritage conservation.

## 4. Hydrogels for Mural Conservation

**Figure 2:**
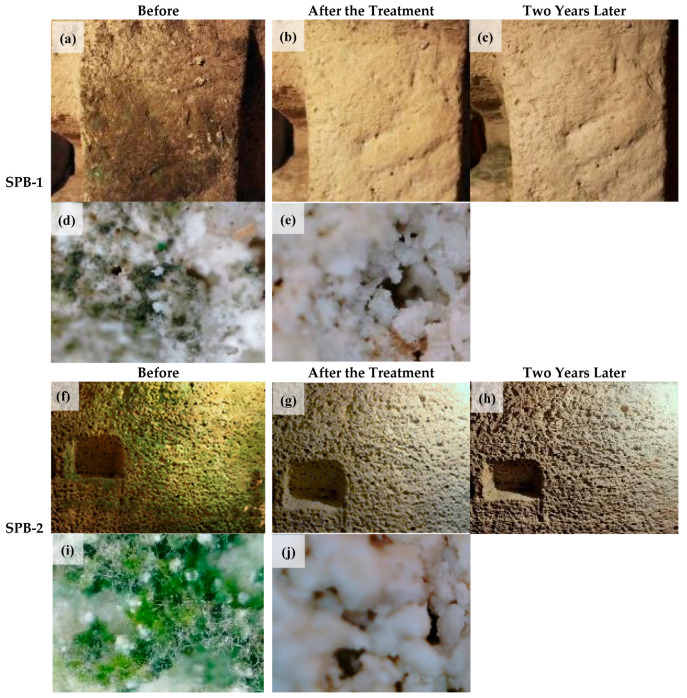
Photographic records of SPB-1 and SPB-2 from the Putridarium in San Pietro Barisano Church—including both standard photographs ((**a**–**c**) for SPB-1; (**f**–**h**) for SPB-2) and 4× magnified digital microscopy images ((**d**,**e**) for SPB-1; (**i**,**j**) for SPB-2)—were captured at three key time points: prior to cleaning (left column), immediately following treatment (middle column), and two years post-cleaning (right column). These images collectively document the visual and microscopic changes in the treated mural surfaces over time. Reproduced with permission [63]. Copyright © 2022, Coatings.

Cleaning murals is an inherently challenging task. Prolonged environmental exposure often leaves their surfaces uneven and degraded, greatly complicating the cleaning process [63]. Common approaches include mechanical removal [64], chemical washing [65], micro-emulsion techniques [66], and hydrogel-based treatments [67]. Among these, hydrogel cleaning has been widely adopted in practice because it is gentle, highly efficient, and causes minimal damage to the mural substrate [68]. Li et al. synthesized a zwitterionic polyelectrolyte, poly (SBMA-co-DMC), by covalently cross-linking sulfobetaine methacrylate with [2-(methacryloyloxy) ethyl] trimethylammonium chloride [69]. This polymer was then incorporated into a poly (vinyl alcohol)/carboxymethyl chitosan hydrogel network to construct a double-network hydrogel with excellent mechanical properties. The resulting hydrogel exhibited tunable adsorption capacity for antimicrobial metal ions (e.g., Cu^2+^) and demonstrated outstanding cleaning performance on mural models. Notably, the hydrogel could adhere stably to rough surfaces, efficiently remove unwanted substances, and leave no detectable residue, offering a safe and effective solution for the cleaning of cultural heritage objects. In the conservation of medieval murals at St. Michael’s Chapel in Barcelona, Spain, applying destructured agar supported by Japanese paper enhanced contact with the uneven mural surface [70]. The addition of tri-ammonium citrate or ethylenediaminetetraacetic acid (EDTA) to the agar gel proved effective in eliminating animal glue residues and fungal stains from the biodeteriorated artwork. On the other hand, to develop an environmentally friendly cleaning material with long-lasting antimicrobial efficacy for mural conservation, researchers formulated a sodium alginate-based biocidal hydrogel incorporating sodium dichloroisocyanurate (1.6 wt.%) and calcium chloride (CaCl_2_, 0.6 wt.%) [38]. Using the “San Pietro Barisano Church” and the “Santa Maria Church” as case studies, the cleaning performance of the hydrogel against microbial biofilms (e.g., fungi and bacteria) on stone wall surfaces was systematically evaluated. The results showed that the hydrogel was able to completely remove biofilms and stains formed by cyanobacteria of the genera Leptolyngbya, Chroococcus, or Gloeocapsa from the surface of church stone materials. Moreover, it provided effective protection for the murals for up to two years (Figure 2). More importantly, the hydrogel reduced the degree of stone powdering by 91%, thereby preserving the structural integrity of the stone to a significant extent.

In recent years, hydrogels loaded with biocidal agents have been widely employed to address biodeterioration in cultural heritage and architectural stone materials. These hydrogels not only significantly reduce the required dosage of biocides, thereby lowering treatment costs, but also enhance environmental sustainability. Building on this approach, Sonaglia et al. developed an ozone-functionalized bacterial cellulose hydrogel and systematically evaluated its efficacy in removing bacteria and fungi from stone surfaces [71]. Mechanical testing results showed that the synthesized hydrogel exhibited a maximum elongation (ε) of 15.5 ± 5.6%, a maximum yield stress (σ) of 18.4 ± 1.1 MPa, and an elastic modulus (E) of 1.5 ± 0.6 MPa, confirming its excellent mechanical properties. Antimicrobial assays demonstrated that after 10 min of treatment, the hydrogel achieved a 90% inhibition rate against *Staphylococcus aureus*. In antibacterial tests against *A. xylosoxidans*, OBC-H treatment caused 90% bacterial death within just 1 min and complete inactivation after 10 min. Observations over 30 days showed that the hydrogel provided sustained inhibition of spores from *Aspergillus aureus*, *Aspergillus xylosoxidans*, and *Aspergillus jensenii*, confirming its significant antifungal and antibacterial efficacy. Simulated stone experiments further revealed that the hydrogel achieved a nearly 100% microbial kill rate within 2 h across all tested samples. In tests evaluating spore germination on biocalcarenite and marble, the hydrogel film exhibited antibacterial activity exceeding 95%, significantly outperforming untreated controls. Additionally, researchers emphasized that all hydrogels could be easily peeled off from the stone surfaces using tweezers without leaving any visible residue. These research findings indicate that hydrogels used for mural conservation have more stringent performance requirements compared to those used for paper-based artifacts. For paper artifacts infected with fungi, a short-term hydrogel treatment is generally sufficient, followed by storage under appropriate conditions once the fungi are removed. In contrast, murals are often exposed to external environments and typically subject to high humidity, which promotes fungal growth and poses challenges for effective protection. Therefore, hydrogels intended for mural conservation must not only possess strong and sustained antimicrobial activity but also maintain structural stability to ensure long-term efficacy. Additionally, such hydrogels should have certain restorative functions to effectively prevent fungal damage while inhibiting stone powdering, thereby preserving the integrity of the murals.

Hydrogels show great potential in mural conservation due to their ability to deliver cleaning and antimicrobial agents safely and effectively, with minimal damage and residue. Recent developments, such as functionalized and double-network hydrogels, have improved adhesion, cleaning efficiency, and long-term antimicrobial protection. However, challenges remain, including residue risk, substrate compatibility, and performance under variable environmental conditions. Future research should focus on material optimization, long-term stability, and tailored formulations for different mural types to ensure safer and more sustainable conservation practices.

## 5. Hydrogels for Ancient Ceramic Conservation

**Figure 3:**
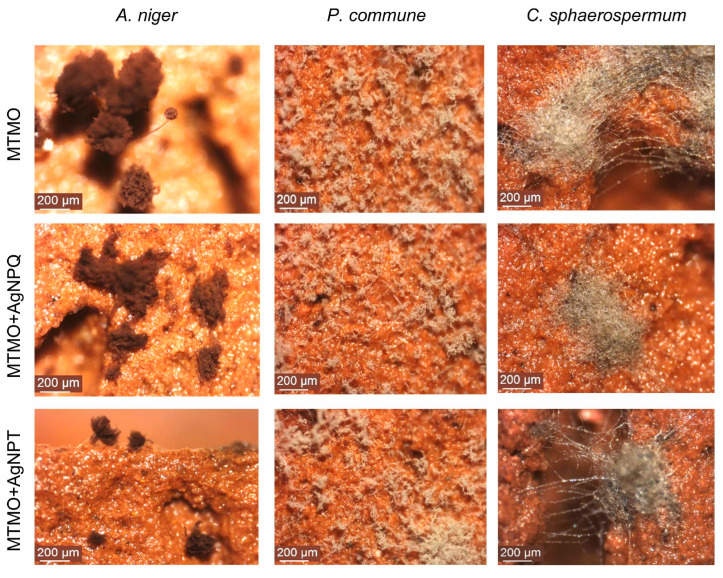
Stereomicroscopic images (80× magnification) showing the fungal resistance performance of sol-gel coatings containing MTMO after 30 days of incubation at 28 °C. Reproduced with permission [66]. Copyright © 2023, Nano-Structures & Nano-Objects.

Ancient ceramics are porous and hygroscopic, making them susceptible to microbial colonization in humid environments [8]. Fungal contamination can lead to aesthetic damage, structural degradation, and long-term deterioration of the ceramic surface [33]. However, current antifungal treatments often lack material compatibility, leave residues, or fail to provide long-lasting protection [33]. In contrast, hydrogels offer several advantages for combating fungal contamination on ancient ceramics, including controlled release of antifungal agents, minimal surface residue, and excellent adaptability to irregular ceramic surfaces, making them a promising solution for safe and effective conservation [72]. For example, Gámez-Espinosa et al. developed antifungal particles through a green synthesis method using tannin-rich aqueous extracts from *Schinopsis balansae* and *Caesalpinia spinosa* [37]. These particles were subsequently incorporated into a hydrogel matrix composed of silver nitrate (AgNO_3_), 3-aminopropyltriethoxysilane, or 3-mercaptopropyltrimethoxysilane, resulting in an antimicrobial hydrogel coating. Compared to the silane + AgNPs treatment, the hydrogel coating exhibited superior antimicrobial activity, completely inhibiting the growth of *A. niger*, *P. commune*, and *C. sphaerospermum*. Additionally, simulated ceramic experiments demonstrated that the hydrogel coating effectively prevented fungal colonization on new ceramic materials and the subsequent formation of fungal biofilms (Figure 3). Moreover, studies have shown that hydrogel coatings containing zinc oxide nanoparticles also exhibit great potential in removing fungal contamination from ceramic surfaces and in the preservation of ceramic cultural heritages [73]. It was able to exhibit satisfactory inhibitory effects against *Penicillium citrinum*, *Trichoderma viride*, and *Aspergillus niger* over a period of one week, with inhibition rates exceeding 50%. At the same time, numerous studies have indicated that hydrogel coatings represent an effective solution for combating fungal infections on ancient ceramic heritage objects [74].

Research on the application of hydrogels for treating fungal contamination on ancient ceramics is still in its early stages. Current studies are limited in number and often focus on general material cleaning rather than ceramic-specific fungal control. In addition, hydrogels designed for antifungal treatment of ceramic cultural heritages must meet a series of specific requirements. First, since ceramic surfaces are typically smooth and hard, and may be coated with decorative pigment layers such as glaze, polychrome painting, or metallic luster, the hydrogel must possess good adhesion and appropriate flexibility to ensure effective coverage without slipping. Compared to murals and paper-based artifacts, ceramics are less sensitive to moisture; however, their surface pigments, especially those affected by long-term weathering or insufficient firing temperatures, can be relatively fragile and susceptible to chemical interference. Therefore, hydrogels intended for ceramic applications must be formulated with mild components and a neutral pH to avoid corrosion or discoloration of painted layers caused by strong acids or bases. Moreover, because ceramics are non-absorbent, antifungal activity must be achieved through surface contact. Thus, the hydrogel should offer sustained release and sufficient contact time, while also being easy to remove completely after application without leaving residues, in order to preserve the original appearance of the ceramic surface and avoid interfering with subsequent conservation treatments.

## 6. Other Important Cultural Relics

### 6.1. Hydrogels for Stone Cultural Relics

**Figure 4:**
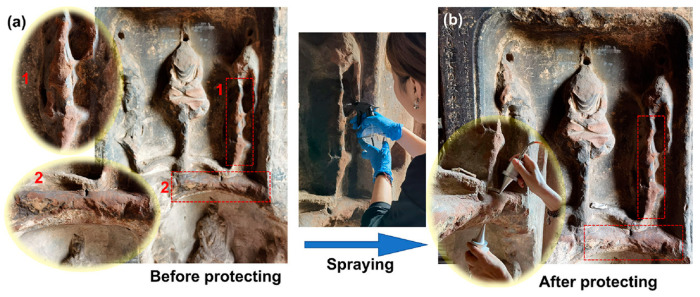
In situ application of a bentonite-based hydrogel for the protection of sandstone cultural heritage at Dafo Temple. Sampling sites are highlighted with red rectangles. (**a**) Surface condition of the sandstone prior to treatment; (**b**) appearance of the sandstone after hydrogel application. Reproduced with permission [42]. Copyright © 2022, ACS Applied Materials & Interfaces.

Stone cultural heritages are often infected by fungi due to long-term exposure to natural environments, leading to surface weathering, discoloration, and even structural damage. Because fungi are highly adaptable and often hidden, conventional chemical treatments can pose risks of secondary damage, making prevention and control challenging. Hydrogel materials, with their excellent controlled-release and adhesion properties, offer a safe and effective new approach for antifungal treatment under mild conditions. Shi et al. recently proposed a novel method for the conservation of sandstone cultural heritage, involving the in situ formation of a bentonite-based hydrogel (BH) within the sandstone matrix [42]. In this study, modified bentonite was successfully incorporated into an acrylamide hydrogel system, and the gelation time and viscosity of BH were effectively controlled by adjusting the reaction parameters. The resulting BH can form within 3 to 5 h, featuring a controllable structure, excellent mechanical properties, and strong resistance to swelling. The BH formed within the sandstone establishes a stable interface with the substrate through multiple interactions, including hydrogen bonding, coordination bonding, ionic bonding, and mechanical interlocking, significantly enhancing the compressive strength of the sandstone. Aging tests demonstrated that the BH-treated sandstone exhibited markedly improved resistance to salt crystallization, acid erosion, and freeze–thaw cycles (Figure 4). Theoretically, this technique can effectively prevent structural loosening of the stone caused by fungal contamination, offering a reliable solution for the long-term preservation of sandstone-based cultural heritages.

### 6.2. Hydrogels for Bone and Keratinous Cultural Relics

Excavated bone and keratin-based artifacts are highly susceptible to environmental changes such as desiccation and microbial invasion. Current preservation technologies generally lack materials that simultaneously provide both moisturizing and antimicrobial functions. To address this issue, researchers have developed a dual-Janus structured polyacrylamide (PAM) hydrogel composite for the hydration and antimicrobial protection of bone artifacts [31]. Mechanical testing demonstrated that the hydrogel possesses excellent adhesion and impact resistance. Studies confirmed that the synergistic effect of PDMS and brominated isobutylene-isoprene rubber (BIIR) significantly enhances the fracture toughness of the composite material, facilitating stable adhesion of the gel on irregularly shaped bone artifacts. Interestingly, the hydrogel exhibited outstanding waterproof performance (WVT: 2343.8 ± 640.2 g/(m^2^·24 h); P_v: 8.5 ± 2.3 × 10^−13^ g·cm/(cm^2^·s·Pa)) as well as good water vapor permeability (WVT: 87.9 ± 38.6 g/(m^2^·24 h); P_v: 1.3 ± 0.6 × 10^−13^ g·cm/(cm^2^·s·Pa)). Additionally, it was found that when the PAM hydrogel is coated with the waterproof BIIR, its water retention ability is significantly improved. The composite hydrogel retained about 90% of its initial weight even after 6 days, demonstrating excellent moisture retention. By incorporating hydrophobic chlorhexidine digluconate (CD) and hydrophilic 4,5-dichloro-2-n-octyl-4-isothiazolin-3-one (DCOIT) into the PDMS and PAM layers, respectively, the hydrogel exhibited superior antimicrobial properties, with in vitro inhibition rates against bacteria and Penicillium approaching 100%. In protection experiments on ivory fragments excavated from the Sanxingdui site, it was observed that unprotected ivory nearly completely lost moisture within about 2 h at room temperature, whereas ivory treated with the BPP composite material maintained over 95% of its mass even after 6 h. Furthermore, antimicrobial tests showed that the control ivory samples were completely covered by mold, while ivory protected by the hydrogel displayed a clear inhibition zone with a mold growth rating of zero, indicating significant antifungal efficacy. This approach provides a promising solution for the long-term preservation of fragile artifacts such as ivory.

### 6.3. Hydrogels for Wooden Cultural Relics

Fungal contamination is a common and serious threat to wooden cultural heritages, especially in humid or poorly ventilated environments. Fungi can degrade cellulose and lignin, leading to structural weakening, surface discoloration, and irreversible damage. Traditional antifungal treatments often lack precision and may introduce harmful chemicals or cause secondary deterioration. In contrast, hydrogels offer a promising alternative by enabling localized, controlled antifungal delivery while maintaining the moisture balance essential for wood preservation. The application of biomass-based hydrogels offers a flexible and eco-friendly solution for the preservation of wooden cultural heritages, demonstrating the great potential of sustainable technologies in both mild intervention and long-term conservation [24]. This approach also simplifies traditional conservation procedures while minimizing environmental impact. For example, researchers developed a self-dissolving hydrogel with both antibacterial and deacidifying functions by incorporating silver nanoparticles (AgNPs) into an alginate/polyacrylamide composite hydrogel, which was successfully applied to the preservation of waterlogged wooden artifacts recovered from the 800-year-old “Nanhai No. 1” shipwreck [24]. The hydrogel exhibits outstanding tensile strength and mechanical robustness, remaining unbroken even at an elongation of up to 2000%, and fully recovering its original shape after stretching. Interestingly, as the silver nanoparticles (AgNPs) undergo changes, the polymer network disintegrates, causing the hydrogel to gradually transition into a solution that releases the antibacterial active agent AgNPs, achieving up to 99% antibacterial efficacy. More importantly, the hydrogel’s self-dissolving property effectively prevents damage to the wood surface during removal, enhancing the safety and convenience of the preservation process.

## 7. Removal Methods of Hydrogels and Their Impact on Cultural Heritages

After completing antifungal treatment, hydrogels should be promptly removed to prevent potential irreversible damage to cultural heritages caused by gel residues. Currently, the removal of hydrogels still relies primarily on physical peeling [75], though in some cases, methods such as rinsing with water, self-dissolution, or chemical/enzymatic degradation have also been employed [24]. Physical peeling is suitable for structurally stable hydrogels like agar and gellan gum [76], which can be lifted off the artifact surface as a whole. This method is simple and leaves minimal residue, but it requires high mechanical integrity of the gel. Water rinsing is typically used for more adhesive materials such as xanthan gum, relying on clean water or buffer solutions to remove residues; however, it is unsuitable for moisture-sensitive artifacts such as paper. In recent years, self-dissolving hydrogels have attracted increasing attention. These materials undergo polymer network disintegration in response to changes in temperature or humidity, gradually transforming into a liquid state while releasing active antifungal agents [24]. This process enables gel removal without mechanical intervention, thereby reducing the risk of surface damage to fragile artifacts. In addition, some studies have explored the use of pH shifts or enzymatic reactions to accelerate gel degradation, offering a more controlled and low-residue removal strategy. Ongoing research aims to develop smarter and safer hydrogel systems. Particularly in the preservation of wooden and paper-based relics, various new-generation hydrogels integrating antifungal, moisturizing, and self-dissolving properties have been proposed. For example, in the conservation of waterlogged wooden artifacts from the “Nanhai No. 1” shipwreck, a self-dissolving alginate/polyacrylamide composite hydrogel was used [24]. This material not only achieved effective antimicrobial and acid-neutralizing functions but also avoided potential structural damage by eliminating the need for manual removal through controlled dissolution. These advances highlight the great potential of self-dissolving and low-residue hydrogels as ideal removal strategies following antifungal treatment in cultural heritage conservation.

## 8. Conclusions and Future Perspectives

Hydrogels have emerged as a promising class of functional materials for the preservation of cultural heritage, particularly in the context of antifungal treatment. Due to their high water content, tunable mechanical properties, and excellent biocompatibility, hydrogels provide a unique platform for the localized and controlled delivery of antimicrobial agents. In particular, their application to wooden, stone, bone, mural, and paper-based artifacts has demonstrated the ability to suppress microbial growth, especially fungal contamination, while maintaining the physicochemical integrity of the original substrates. This is of great importance, as fungal colonization is a major contributor to biodeterioration, often resulting in irreversible structural damage, discoloration, and the loss of surface information essential for historical and aesthetic value.

Despite these advantages, the application of hydrogels for antifungal treatment in cultural heritage remains in its infancy. To date, only a limited number of case studies have explored their use in real-world conservation scenarios. Most existing research has been conducted under controlled laboratory conditions or focused on proof-of-concept models. Moreover, while materials such as silver nanoparticles, essential oils, or chlorhexidine have been incorporated into hydrogel matrices for antimicrobial purposes, the long-term effects of these agents on both the microbial community and the artifact material remain insufficiently understood [76]. Moreover, current research on the drug release mechanisms of hydrogels used in cultural heritage preservation remains limited. Most studies have primarily focused on their practical performance, such as antimicrobial efficacy, with insufficient attention given to the underlying characteristics and modes of drug release. For example, it remains unclear whether the observed antimicrobial activity results from direct contact killing or sustained release of active agents, critical questions that have yet to be systematically explored. In situ degradation, residue removal, and interaction with complex artifact surfaces all represent practical challenges that must be addressed. These limitations highlight the need for interdisciplinary collaborations between materials scientists, microbiologists, and heritage conservators to refine hydrogel formulations and application protocols tailored to the diverse nature of cultural heritages.

Beyond their antifungal potential, hydrogels have also shown great promise in broader conservation functions, particularly in cleaning, stabilization, and restoration processes. Their unique viscoelasticity and solvent retention capacity allow for the gentle and precise removal of surface contaminants such as soot, salts, and aged varnishes, minimizing the risk of mechanical or chemical damage to fragile substrates. Similarly, in waterlogged archaeological wood, hydrogel-based systems have been used to deliver consolidants or pH-regulating agents in a controlled manner, ensuring uniform treatment while avoiding local overexposure.

The multifunctional potential of hydrogels opens exciting opportunities for the next generation of conservation materials. Future research should focus on designing smart hydrogel systems capable of integrating multiple functions, such as antimicrobial activity, pH buffering, pollutant capture, and mechanical reinforcement, into a single, synergistic platform. Responsive hydrogels that adapt their properties based on environmental cues (e.g., humidity, pH, or microbial load) could enable real-time, self-regulating conservation treatments. Moreover, the development of biodegradable or self-dissolving hydrogel formulations would eliminate the need for invasive removal procedures, reducing the risk of secondary damage and simplifying post-treatment protocols.

From a sustainability perspective, emphasis should also be placed on the use of renewable, non-toxic biopolymers, such as chitosan, alginate, cellulose derivatives, and gelatin, as the basis for hydrogel design. These materials not only align with green chemistry principles but also exhibit favorable interactions with natural artifact substrates. Combined with low-cost, scalable fabrication techniques such as 3D printing or in situ gelation, hydrogel technologies hold the potential to democratize advanced conservation methods for institutions with limited resources. In addition, certain important artifacts, such as textiles, also require the development of highly effective protective hydrogels specifically suited to their preservation needs.

In summary, hydrogels represent a highly versatile and underexplored class of materials for cultural heritage conservation, particularly in the realm of fungal prevention and antimicrobial treatment. While existing studies have demonstrated proof of concept and strong practical potential, their application remains limited, and significant research is still required to bring hydrogel technologies to the forefront of mainstream conservation practice. By expanding their functionality beyond disinfection into areas such as structural stabilization, cleaning, and environmental buffering, hydrogels may pave the way toward integrated, multifunctional preservation systems that align with both the ethical and scientific standards of modern conservation. Continued efforts in this direction will not only enhance our ability to safeguard cultural heritages from biodeterioration but also promote the development of sustainable, effective, and minimally invasive conservation solutions for future generations.

## Figures and Tables

**Table 1:** Classification of major fungi infecting cultural heritages and their mechanisms of deterioration.

Major Fungal Taxon		Key Deterioration Materials	Main Degradation Mechanisms	References
*Ascomycota*	*Aspergillus* spp.	Paper, Murals, Textiles, Leather, Ancient Ceramics, Stone (covering nearly all kinds of biodeteriorated objects of cultural heritage)	1. Organic cultural materials (paper, textiles, leather, etc.) (1) Enzymatic degradation (primary mechanism): Fungi secrete extracellular enzymes to decompose organic components in cultural heritage. (2) Acidic metabolites. (3) Discoloration and Metabolite Contamination. 2. Inorganic cultural materials (murals, stone artifacts, metals) (1) Biogenic acid erosion: Secretes organic acids to dissolve carbonates (e.g., CaCO_3_ in murals). (2) Biofilm disruption. (3) Metal corrosion. 3. Synergistic environmental effects (1) Humidity-dependent activity. (2) Microbial symbiosis(e.g., enhanced degradation through bacterial synergism).	[8,34,35,36,37]
	*Penicillium* spp.			
	*Cladosporium* spp.			
	*Fusarium* spp.			[35,38]
	*Alternaria* spp.			[35]
	*Trichoderma* spp.			[35,38,39]
	*Chaetomium* spp.			[35,37]
*Zygomycota*	*Mucor* spp.	Textiles, Paper, Ancient Ceramics	1. Physical biofilm formation. 2. Mild organic acid secretion.	[8]
*Basidiomycota*	Brown (e.g.: *Serpula lacrymans*)—and white-rot (e.g.: *Schizophyllum commune*) decay fungi	Wood (highly specialized)	Decompose and utilize lignin, cellulose, and hemicellulose.	[24,40,41]
	Lichens (a symbiotic partnership between a fungus and a phototrophic organism)	Stone Monuments, Buildings, Cement and Mortar	1. Chemical degradation (Dominant Mechanism) (1) Organic acid corrosion. (2) Chelation. 2. Physical mechanical deterioration 3. Biomineralization deposition	[33,35,42]

## Data Availability

No new data were created.

## Abbreviations

The following abbreviations are used in this manuscript:

semi-IPN	semi-interpenetrating network
PVA	Poly(vinyl alcohol)
PHEAA	Poly(N-(2-hydroxyethyl)acrylamide
SGs	Soft gels
RGs	Rigid gels
EDTA	Ethylenediaminetetraacetic acid
BH	Bentonite-based hydrogel
PAM	Polyacrylamide
AgNPs	Silver nanoparticles
